# Supplementary material for: Robotic agricultural instrument for automated extraction of nematode cysts and eggs from soil to improve integrated pest management
Source: Sci Rep. 2021 Feb 5;11:3212. doi: 10.1038/s41598-021-82261-w (PMC7864952; doi:10.1038/s41598-021-82261-w)
Supplement: Supplementary file 1 — Supplementary Information. [file 41598_2021_82261_MOESM1_ESM.docx]

Supplementary Materials for:

**Robotic agricultural instrument for automated extraction of nematode cysts and eggs from soil to improve integrated pest management**

**Authors**

Christopher M. Legner^1^, Gregory L. Tylka,^2^ Santosh Pandey^1^*

**Affiliations**

^1^Department of Electrical & Computer Engineering, Iowa State University, Ames, Iowa, USA,

^2^Department of Plant Pathology and Microbiology, Iowa State University, Ames, Iowa, USA, *Corresponding author. Email: [pandey@iastate.edu](mailto:pandey@iastate.edu)

**This PDF file includes:**

Fig. S1. Manual wet-sieving extraction technique

Fig. S2. Control electronics

Fig. S3. Drainage system

Fig. S4. User interface software

**Other Supplementary Material for this manuscript includes the following:**

Movie S1 (.mp4 format). Rotational motion of the stage levels

Movie S2 (.mp4 format). Vertical raising and alignment of the stage levels

Movie S3 (.mp4 format). Sieve manipulation using the gripper armature

Movie S4 (.mp4 format). Grinding and washing operations to rupture cysts

Movie S5 (.mp4 format). Overall process flow of the instrument (at 2× speed)


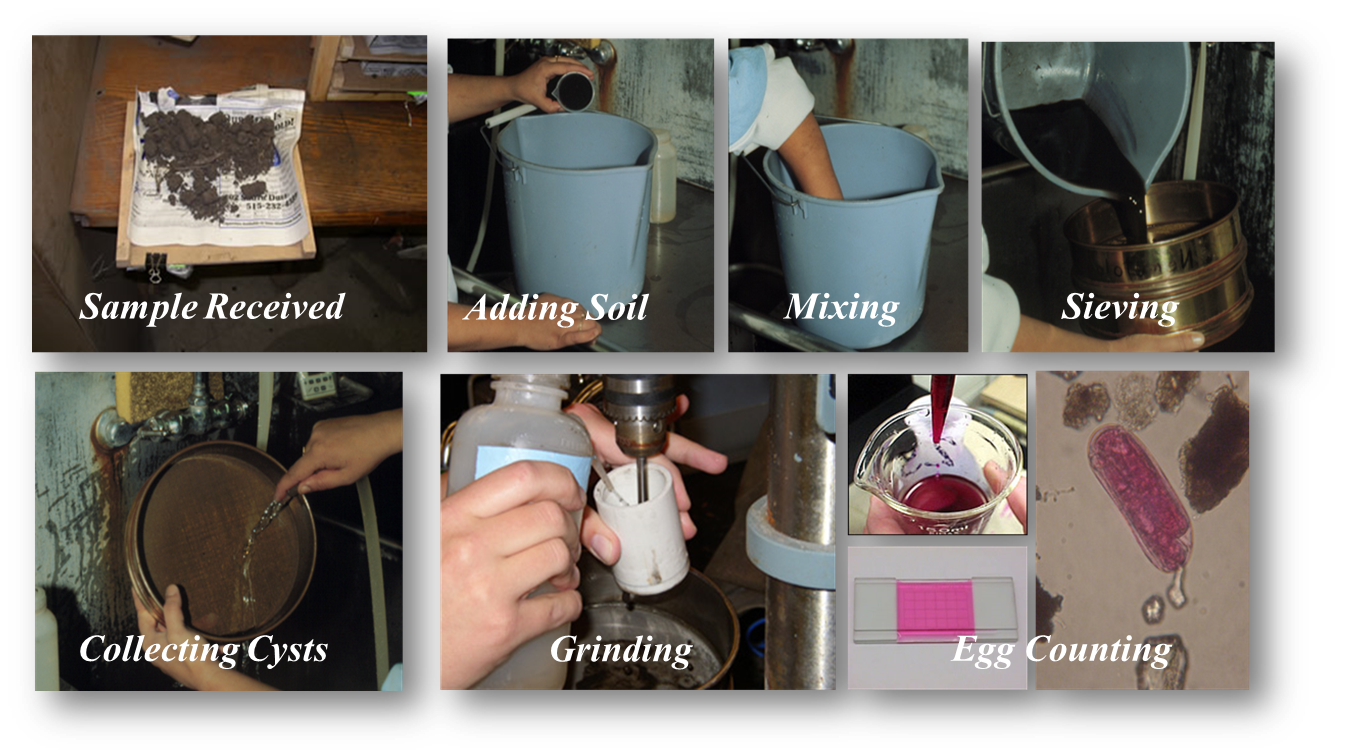


**Fig. S1. Manual wet-sieving extraction technique.** The conventional method of wet-sieving to extract nematode cysts and eggs involves mixing and suspending a soil sample in water, letting heavy soil particles settle out, sieving water containing suspended sediments and cysts, collecting and counting cysts, if desired, or grinding the cysts to extract eggs followed by sieving egg suspension to recover and count eggs.

Image credit: <https://www.plantpath.iastate.edu/scn/protocols_extractcysts>

***
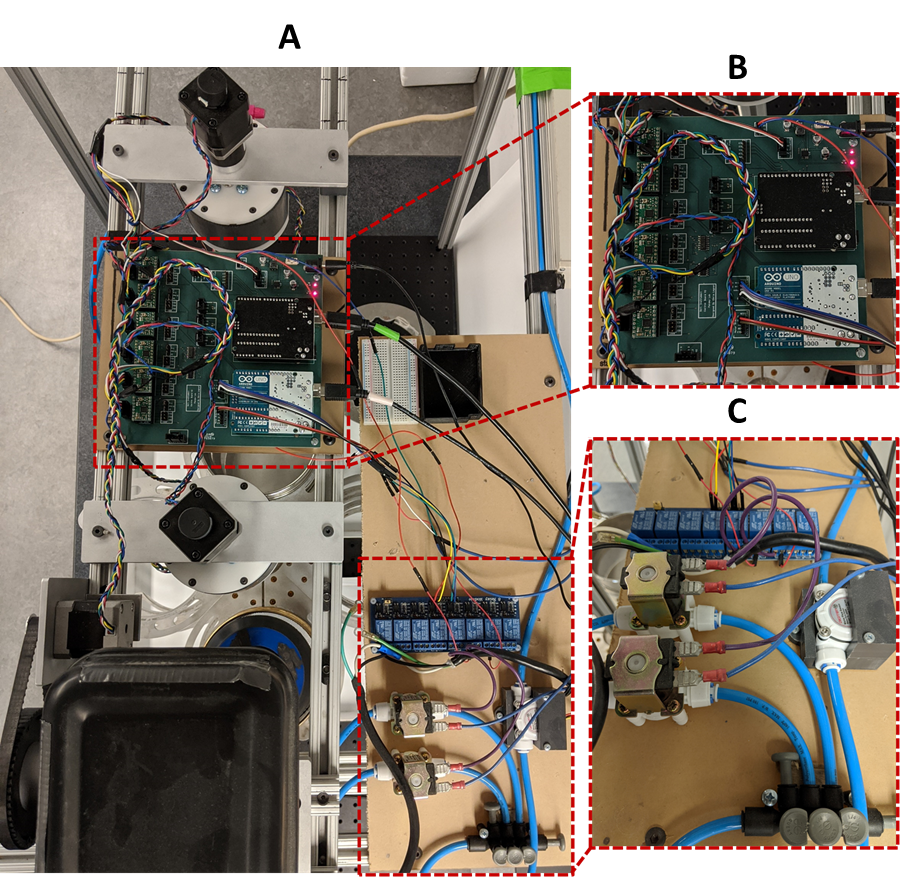
***

**Fig. S2. Control electronics.** (**A**) Top-down view of the robotic instrument showing the control electronics system. (**B**) The control board contains two microcontroller units and six stepper driver modules. (**C**) The off-board electronics includes a flowmeter, multichannel relay module, and two solenoid valves.

*
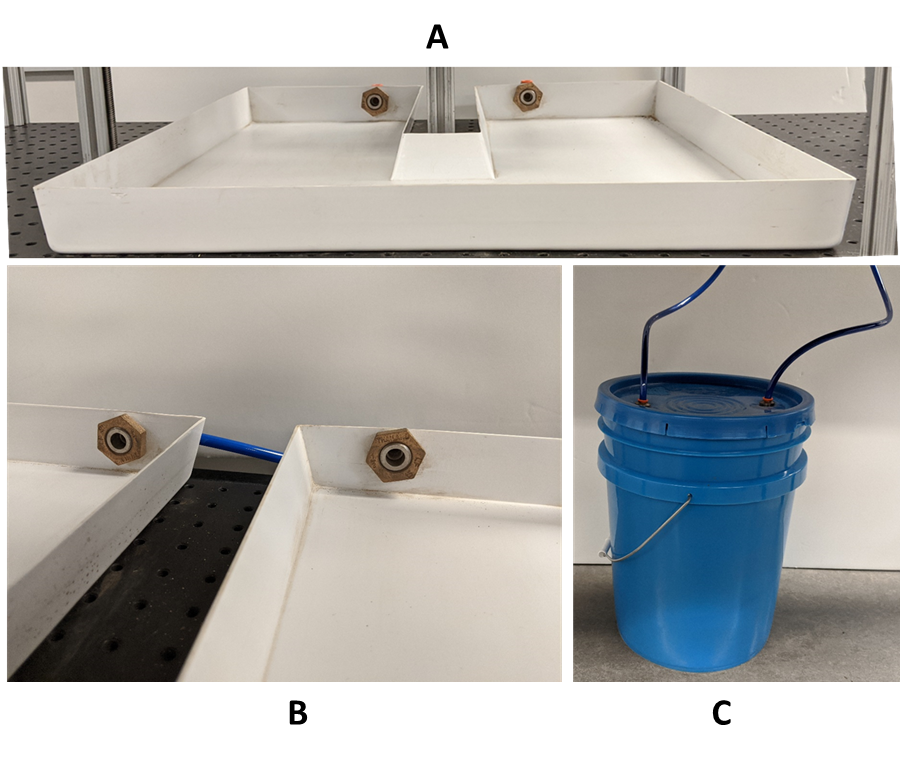
*

**Fig. S3. Drainage system.** (**A**) Removable catch pan is placed beneath the stage system to collect the water and debris after the wet-sieving process. (**B**) Raised drainage outlets is fitted into the catch pan to drain the collected water and remove sediments. (**C**) Waste reservoir collects the residual and dirty water for eventual disposal after multiple runs. The lid can be conveniently lifted to empty the waste reservoir without any need to remove the hose connections.


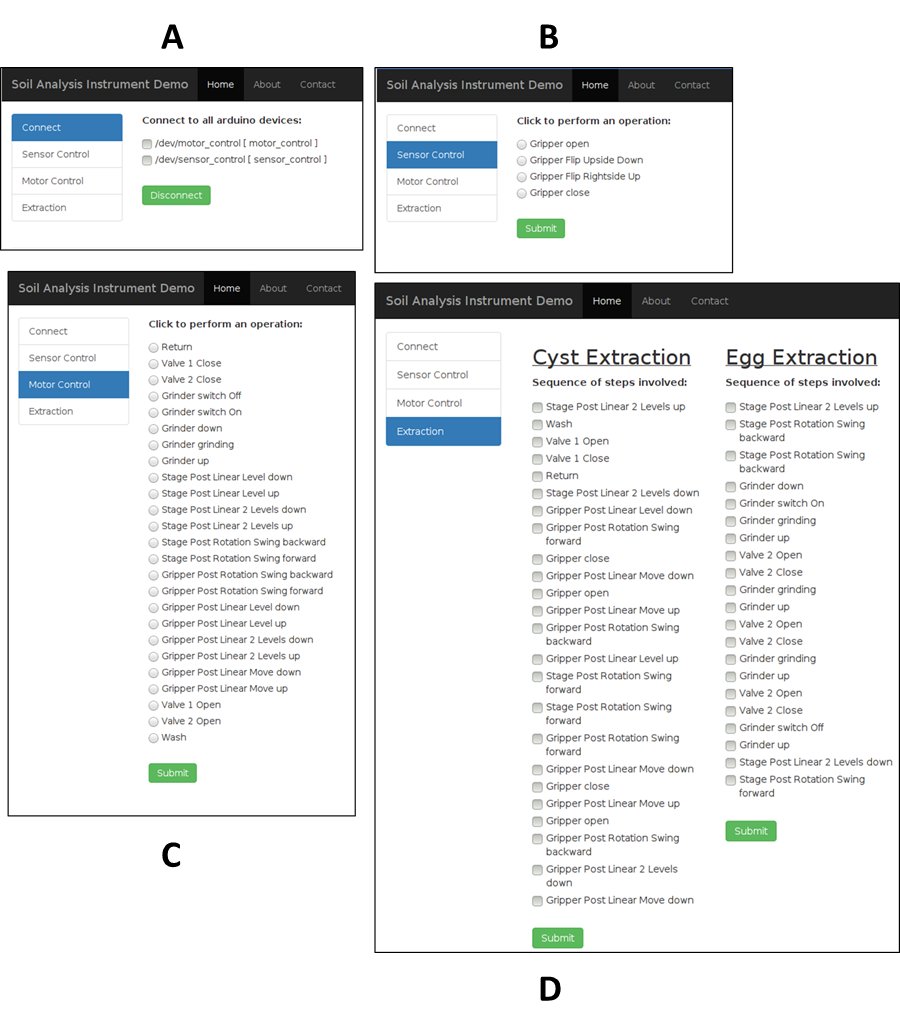


**Fig. S4. User interface software.** All the operations of the robotic instrument are displayed on four GUI screens: Connect, Sensor Control, Motor Control, and Extraction. (**A**) The ‘Connect’ screen initiates communication with the control electronics. (**B**) The ‘Sensor Control’ screen actuates the gripping functions of the gripper. (**C**) The ‘Motor Control’ screen actuates the motion and valving of the robotic instrument. (**D**) The ‘Extraction’ screen performs a set of functions related to the nematode cyst or egg extraction protocols.
